# Two Cases of Primary Testicular Lymphoma Presenting with Direct Spread along the Spermatic Cord and Gonadal Vessels

**DOI:** 10.1155/2019/5953618

**Published:** 2019-06-18

**Authors:** Mostafa Ellatif, Raekha Kumar, Alexander Weller, David Katz, Eirini Vrentzou

**Affiliations:** Department of Radiology, Northwick Park Hospital, Watford Road, London HA1 3UJ, UK

## Abstract

Primary testicular lymphoma is a rare testicular neoplasm that mainly affects elderly patients, with Human Immunodeficiency Virus (HIV) being a known risk factor in the younger population. Approximately 20% of patients will have disseminated disease with extra-nodal involvement at clinical presentation. Rarely, direct spread along the spermatic cord and gonadal vessels can occur and has been described in the literature. We present two cases of this phenomenon where the primary testicular tumour has spread along the gonadal vein to its origin at the inferior vena cava.

## 1. Background

Primary Testicular Lymphoma (PTL) is an uncommon neoplasm representing less than 5% of all testicular tumours and between 1-2% of all cases of non-Hodgkin's Lymphoma (NHL) [1]. Diffuse large B-cell lymphoma (DLBCL) is the most common histological subtype, but T-cell and Burkitt lymphoma have also been described [2]. It is the most common testicular neoplasm in men over the age of 60 and most common bilateral testicular neoplasm [3, 4]. Alongside age, HIV is the best-described risk factor causing an increase in incidence of extra-nodal NHL in younger patients [3].

PTL typically presents as a unilateral painless testicular mass or scrotal swelling and is difficult to differentiate clinically and on imaging from germ cell tumours. Rarely pain may occur, while constitutional symptoms are indicative of systemic disease, which is present in 20% of patients at the time of diagnosis.

NHL of the testis may be associated with extra-nodal involvement of the central nervous system (CNS), Waldeyer's ring, lungs, pleura, skin, and soft tissues as well as the contralateral testis. A small number of case reports however describe direct spread along the spermatic cord and gonadal vessels into the retroperitoneum [5]. In the two biopsy confirmed cases of PTL presented below, staging Computed Tomography (CT) scans identified contiguous disease extension from an enlarged testis along the spermatic cord and gonadal vessels, in addition to sporadic nodal and extra-nodal disease deposits elsewhere in the body. Although rare, this homogeneous cord-like soft tissue extension through the inguinal canal and along the retroperitoneum to the inferior vena cava helps differentiate PTL from other testicular lesions as it has only ever been described with the former [6].

## 2. Case One

A previously well 77-year-old gentleman presented with a 6-week history of right-sided testicular swelling and gradual onset of pain with no preceding history of trauma or known malignancy. He was initially treated in primary care for suspected orchitis but due to persisting symptoms he was referred for a scrotal ultrasound.

**Figure 1:**
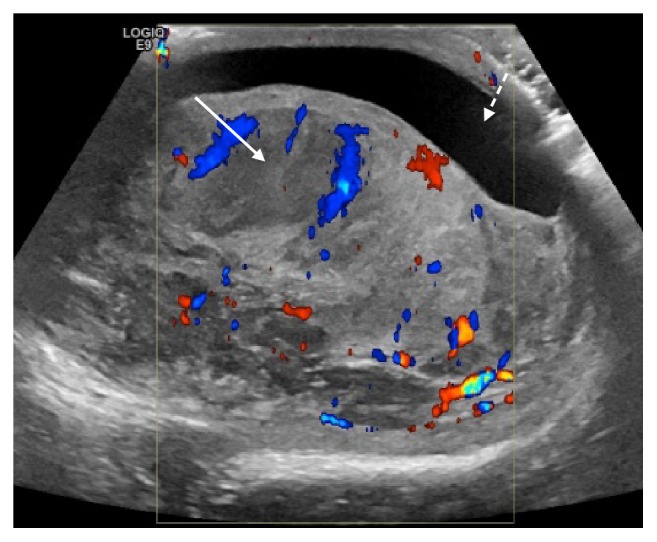
Ultrasound of the scrotum demonstrates an enlarged, heterogeneous, hypervascular right testicle and epididymis with focal, relatively hypovascular hypoechoic areas in the testicular parenchyma (white arrow) and an associated small hydrocele (dashed white arrow).

The ultrasound study (Figure 1) demonstrated a diffusely enlarged, heterogeneous, hypervascular right testicle with two more discrete hypoechoic intraparenchymal lesions showing minimal internal vascularity and a small associated hydrocoele. The ipsilateral epididymis and spermatic cord also appeared diffusely enlarged and heterogeneous with contiguous involvement of the spermatic cord. As suspicion regarding malignancy was high, with lymphoma the working diagnosis due to age, a staging CT of the neck, chest, abdomen and pelvis was arranged.

**Figure 2:**
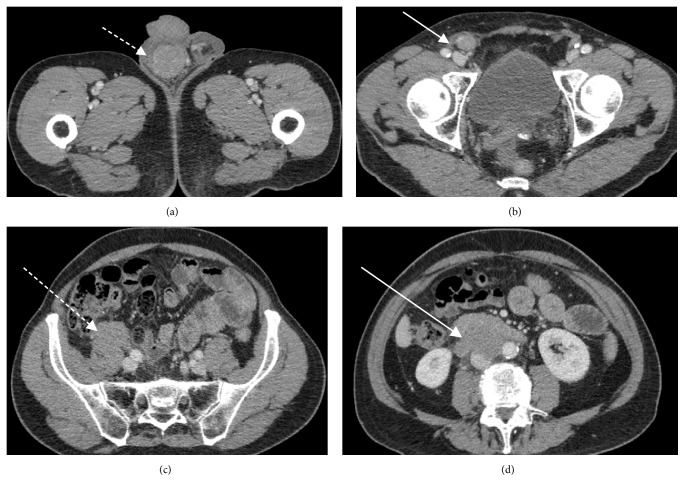
(a) Axial CT image with contrast at the level of the scrotum demonstrates a right testicular mass (dashed white arrow). (b) Axial CT image with contrast at the level of the inguinal canal demonstrates superior tumour infiltration along the right spermatic cord (white arrow). (c) Axial CT image with contrast at the level of the common iliac vessels demonstrates tumour extension along the right gonadal vein (dashed white arrow). (d) Axial CT image with contrast at the level of the kidneys demonstrating confluent aortocaval soft tissue at the site of gonadal vein insertion into the IVC (white arrow).

**Figure 3:**
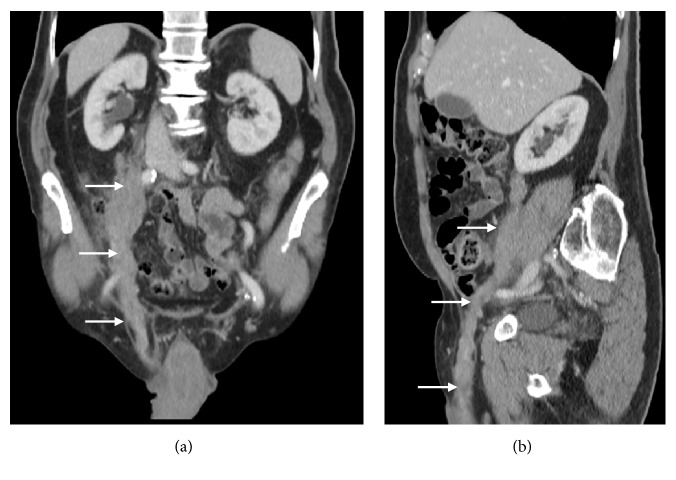
(a and b) Coronal and sagittal reformatted CT images of the abdomen and pelvis with contrast which demonstrate cord-like soft tissue extension from the right testicle into the retroperitoneum along the spermatic cord and gonadal vein.

**Figure 4:**
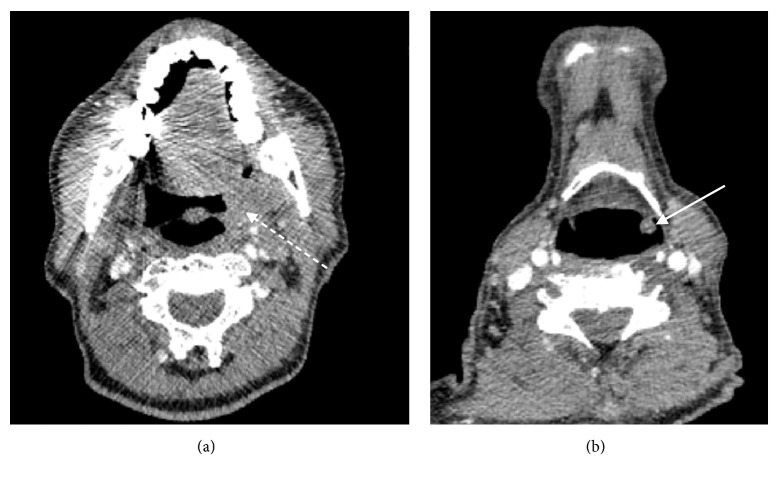
(a) Axial CT image with contrast at the level of the suprahyoid neck revealing an enlarged left palatine tonsil (dashed white arrow). (b) Axial CT image with contrast at the level of the hyoid bone which demonstrates a subcentimetre mucosal soft tissue nodule in the left aryepiglottic fold (white arrow).

CT demonstrated an enhancing right-sided testicular mass (Figure 2(a)) with soft tissue extending along the spermatic cord (Figures 2(b)–2(d)), through the inguinal canal and cranially in the retroperitoneum along the gonadal vein to the level of its insertion into the inferior vena cava (Figures 3(a)-3(b)), locally forming a confluent mass. In addition, an enlarged left faucial tonsil (Figure 4(a)), a mucosal soft tissue nodule (Figure 4(b)) in the left aryepiglottic fold and bilateral adrenal lesions were identified.

Following a multidisciplinary team (MDT) discussion and with lymphoma being the main differential due to the distribution of the lesions, the testicular mass was biopsied under ultrasound guidance and histology results demonstrated diffuse large B-cell lymphoma (germinal centre subtype). Lymphomatous tonsillar involvement was confirmed on biopsy and gastroscopy following an episode of haematemesis showed gastric infiltration, not evident on imaging. Imaging investigations were completed with whole spine and brain Magnetic Resonance Imaging (MRI) to assess for CNS involvement. The patient was subsequently commenced on chemotherapy for stage VI Diffuse large B-cell lymphoma, with follow-up 3-month imaging showing very good partial response.

## 3. Case Two

An 82-year-old patient with a background of monoclonal gammopathy of undetermined significance (MGUS) and previous prostate cancer treated with external beam radiation therapy presented with constitutional symptoms. On clinical examination an enlarged right testicle was noted and serum biochemistry revealed hypercalcaemia.

**Figure 5:**
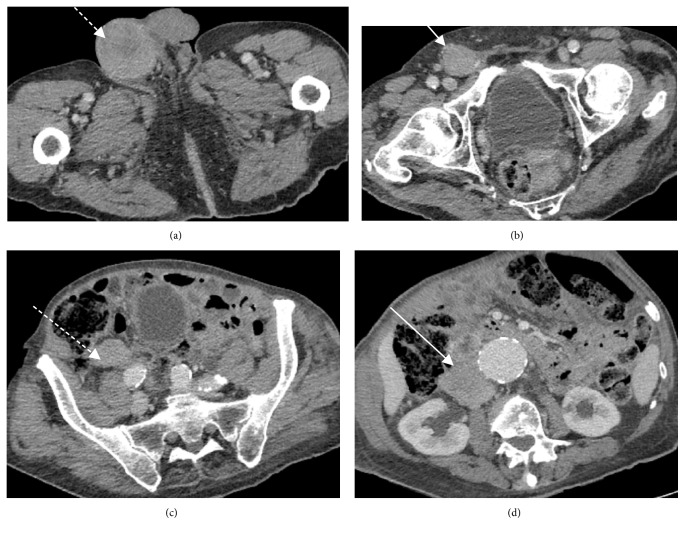
(a) Axial CT image with contrast at the level of the scrotum demonstrates a large, heterogeneous, right testicular tumour (dashed white arrow). (b) Axial CT image with contrast at the level of the right inguinal canal demonstrates extension of homogeneous soft tissue along the right spermatic cord in the inguinal canal (white arrow). (c) Axial CT image with contrast at the level of the common iliac vessels demonstrates tumour anterior to the right iliac artery and vein (dashed white arrow) along the course of the right gonadal vein. (d) Axial CT image with contrast at the level of the right renal pelvis demonstrates soft tissue in the retroperitoneum along the right gonadal vein to the level of its insertion into the inferior vena cava (white arrow).

**Figure 6:**
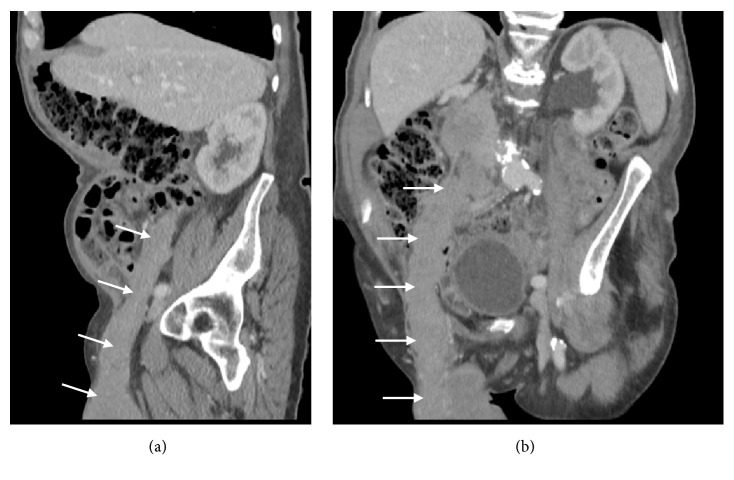
(a and b) Sagittal and coronal reformatted CT images of the abdomen and pelvis with contrast demonstrating cranial extension of the tumour from the scrotum, along the spermatic cord in the inguinal canal and into the retroperitoneum along the right gonadal vein, to the level of its insertion into the inferior vena cava. Incidental note made of a left extrarenal pelvis.

**Figure 7:**
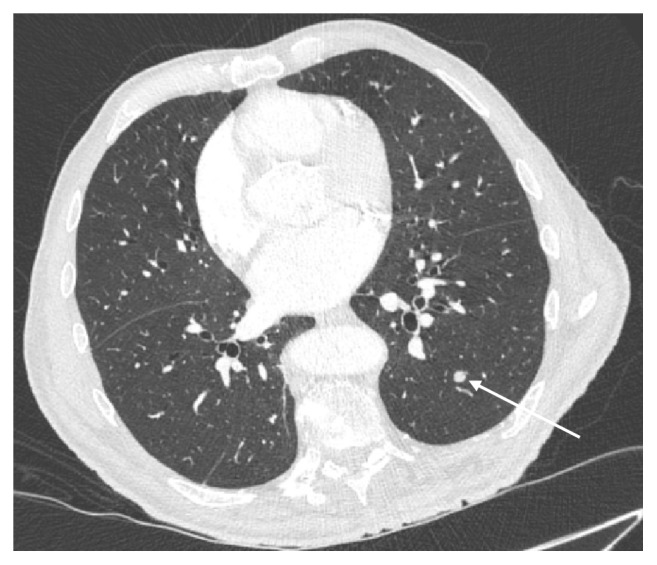
Axial CT image of the chest with lung windows revealing a pulmonary nodule in the posterior basal segment of the left lower lobe (white arrow).

A CT of the chest, abdomen, and pelvis was performed to assess for a new underlying malignancy or prostate cancer recurrence. The study demonstrated a large right-sided scrotal mass (Figures 5(a)–5(d)) with soft tissue extending through the inguinal canal and along the right gonadal vein throughout its course to the insertion point into the inferior vena cava (Figures 6(a)-6(b)), as well as a few bilateral lung nodules measuring up to 14mm, considered to be metastatic (Figure 7).

**Figure 8:**
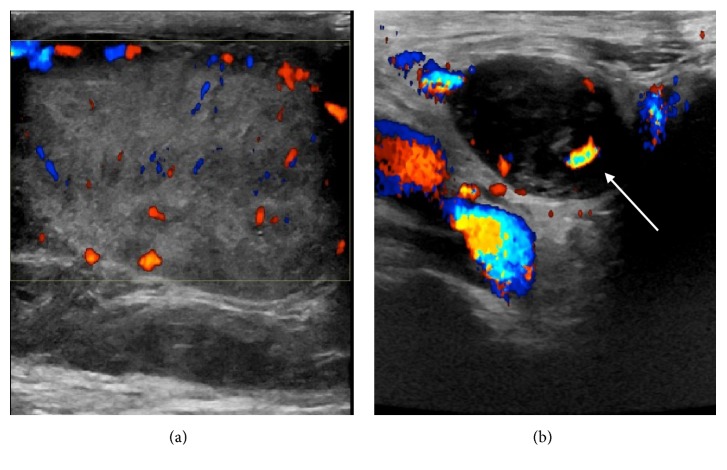
(a) Ultrasound of the scrotum demonstrates an enlarged diffusely heterogeneous right testicle and epididymis with areas of increased vascularity. (b) Ultrasound of the right spermatic cord at the level of the right inguinal canal. Image demonstrates a tubular predominantly hypoechoic mass with internal vascularity (white arrow).

A subsequent ultrasound (Figure 8) was performed to further assess the scrotal lesion. This showed a heterogeneous mass replacing the right testicle, with mass like soft tissue infiltration of the right epididymis and spermatic cord, demonstrating increased Doppler vascularity.

After discussion at the urology cancer MDT a differential diagnosis of sarcoma and lymphoma was suggested and a decision for ultrasound guided biopsy of the testicular lesion was made, rather than orchiectomy due to epididymal and spermatic cord involvement. Histology was consistent with diffuse large B-cell lymphoma, germinal centre subtype. The patient was subsequently referred to haematology and following 3-cycles of chemotherapy demonstrated complete radiological response.

## 4. Discussion

As PTL carries a worse prognosis (5-year survival rate of approximately 12%) than either germ cell tumours or NHL involving lymph-node groups or other extra-nodal sites, timely differentiation from other scrotal masses is required [7]. Treatment options vary but often involve orchiectomy for therapeutic and in some cases diagnostic purposes [8, 9].

The main differential diagnoses for PTL include germ cell tumours, acute and chronic epididymo-orchitis and leukaemia. Unlike lymphoma, germ cell tumours are frequently associated with raised serum Human chorionic gonadotropin (HCG) or alpha-fetoprotein (AFP) and metastasise in a predictable fashion cranially through successive retroperitoneal lymph-node groups [10]. Although infection usually presents as painful scrotal enlargement, with testicular and epididymal hypervascularity at ultrasound, chronic epididymo-orchitis can manifest as an infiltrative scrotal mass occasionally requiring orchiectomy for diagnosis [11]. Typical sonographic features of PTL vary between an enlarged, diffusely hypoechoic and hypervascular testis; unilateral or bilateral focal hypoechoic nodules; or a striated testicular pattern. The tunica albuginea and epididymis can be involved and a hydrocele occurs in approximately 50% of cases [5, 10]. However, none of these features in isolation is specific to PTL, meaning diagnostic biopsy is required.

At CT and ultrasound, sporadic nodal, and extra-nodal deposits around the body increase the probability of lymphoma over germ cell tumours or infection. As in the two cases presented here, cord-like soft tissue extending in a contiguous fashion from the testicular mass onto spermatic cord and gonadal vessels in the retroperitoneum is a very specific feature that has only ever been described in PTL.
